# Cangrelor in a challenging scenario of concomitant ischaemic stroke, pulmonary embolism, and ST-elevation myocardial infarction: a case report

**DOI:** 10.1093/ehjcr/ytae066

**Published:** 2024-01-30

**Authors:** Federico Oliveri, Lorenzo Tua, Rita Camporotondo, Valeria Gritti, Sergio Leonardi

**Affiliations:** Division of Cardiology, University of Pavia, Via Strada Nuova, 65, 27100 Pavia PV, Italy; Department of Cardiology, Leiden University Medical Center, Albinusdreef 2, 2333 ZA, Leiden, The Netherlands; Division of Cardiology, University of Pavia, Via Strada Nuova, 65, 27100 Pavia PV, Italy; Division of Cardiology, Fondazione IRCCS Policlinico San Matteo, Pavia, Italy; Division of Cardiology, Fondazione IRCCS Policlinico San Matteo, Pavia, Italy; Division of Cardiology, University of Pavia, Via Strada Nuova, 65, 27100 Pavia PV, Italy; Division of Cardiology, Fondazione IRCCS Policlinico San Matteo, Pavia, Italy

**Keywords:** Cangrelor, Acute ischaemic stroke, Pulmonary embolism, STEMI, Ischaemic risk, Bleeding risk, Case report

## Abstract

**Background:**

Antithrombotic therapy in acute patients with both high ischaemic and bleeding risks remains challenging.

**Case summary:**

We presented a challenging case involving a 48-year-old man referred to our hospital for headache and a left superior quadrantanopia. A CT scan revealed a right inferior occipital lobe ischaemic stroke. During the hospital stay, the patients developed pulmonary embolism (PE), and ST-elevation myocardial infarction (STEMI). A triple antithrombotic therapy was indicated, but the patient presented with high bleeding (anaemia, active malignancy, ischaemic stroke) and ischaemic (ischaemic stroke, PE, and superimposed STEMI) risks. In this critical acute setting, prolonged cangrelor infusion of reduced dosage, coupled with aspirin and enoxaparin, proved an effective and safe antithrombotic approach.

**Discussion:**

Prolonged cangrelor bridging at a reduced dose of 0.75 μg/kg/min may represent an effective and safe option in acute patients requiring P2Y12 inhibition and presenting both high ischaemic and high bleeding risks.

**Learning points**

- To expand the indications for cangrelor in acute patients exhibiting both high ischaemic and bleeding risks.
- To meticulously balance ischaemic and bleeding risks in acute scenarios.
- To rigorously monitor clinical and imaging findings to guide therapeutic decisions in acute settings marked by high ischaemic and bleeding risks.

## Introduction

The management of antithrombotic therapy after percutaneous coronary intervention (PCI) depends on multiple factors, such as the clinical scenario (acute or chronic), comorbidities (e.g. atrial fibrillation), and the ischaemic/haemorrhagic risk profile of the patient.^1^ However, real-world scenarios can be challenging, and a more ‘tailored’ therapy is advisable. For example, major guidelines are less clear in scenarios with both high ischaemic and haemorrhagic risks. In these patients, especially in acute settings, balancing the need for effective antithrombotic therapy with the risk of severe bleeding is difficult. Cangrelor, with its pharmacokinetic and pharmacodynamic properties (rapid and potent antithrombotic effects coupled with a quick reversal mechanism), might offer a valuable tool in these acute and challenging situations.^2,3^ The presented case involves a patient experiencing concomitant ischaemic stroke, pulmonary embolism, and anterior ST-elevation myocardial infarction (STEMI) within a 24 h period. In addition to these life-threatening conditions, the challenge of navigating between ischaemic and haemorrhagic risks had to be addressed.

## Summary figure

**Table:** 

Time	Events
Presentation	The patient arrived at the ER with complaints of a headache and visual impairment since the day before. Physical examination revealed an isolated left superior quadrantanopia. A brain CT scan confirmed the presence of an (subacute) ischaemic stroke. Aspirin was started.
Day 1	A chest and neck CT with contrast revealed incidental evidence of pulmonary embolism and pulmonary nodule. Enoxaparin 8000 UI b.i.d. was started.
Day 2	The patient experienced crushing chest pain and diaphoresis. The electrocardiogram was compatible with antero-lateral ST-elevation myocardial infarction.
Day 2	PCI of left anterior descending artery (LAD) was completed. Cangrelor was initiated, starting with a bolus of 30 μg/kg, followed by a maintenance dose of 4 μg/kg/min until the end of the procedure. This was then reduced to 0.75 μg/kg/min for up to 12 h post-procedure.
Day 3	A repeated brain CT scan showed no haemorrhagic transformation of the stroke or new ischaemic lesions. The administration of cangrelor was discontinued (after a total infusion time of 12 h), and clopidogrel was initiated.
Day 7	Pancreatic cancer was diagnosed. Subsequently, chemotherapy was begun and the patient was discharged with a treatment regimen of triple antithrombotic therapy (aspirin + clopidogrel + enoxaparin).
Day 30	At follow-up, no ischaemic or bleeding events occurred. Aspirin was discontinued, clopidogrel 75 mg was maintained, and oral apixaban was initiated (10 mg b.i.d. for the first 7 days, followed by a maintenance dose of 5 mg b.i.d.

## Case presentation

**Figure 1:**
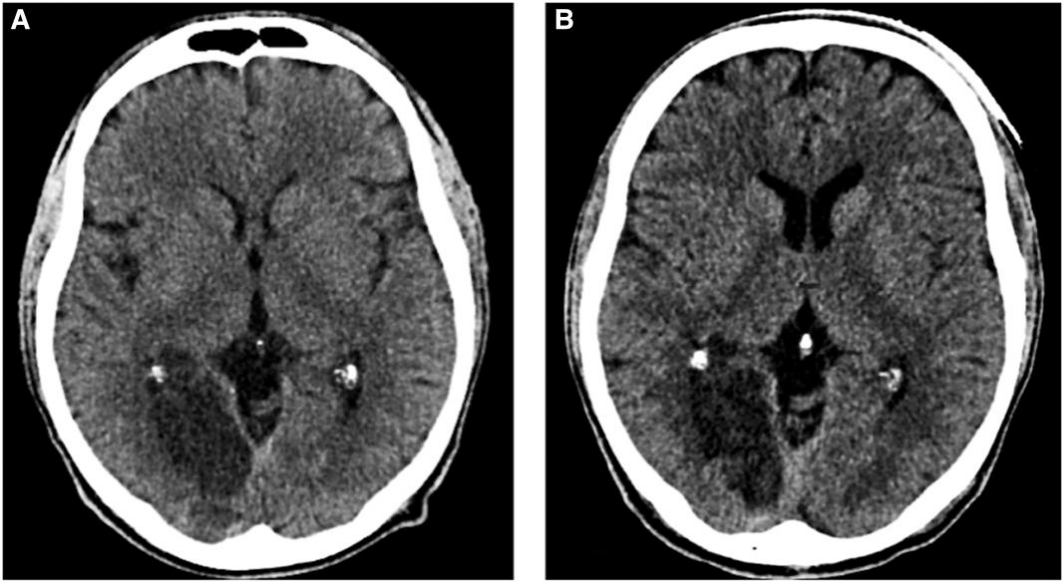
Brain CT scan without contrast performed in the emergency department (*A*) and 12 h after PCI (*B*). No bleeding transformation of the ischaemic stroke was assessed during the cangrelor infusion.

**Figure 2:**
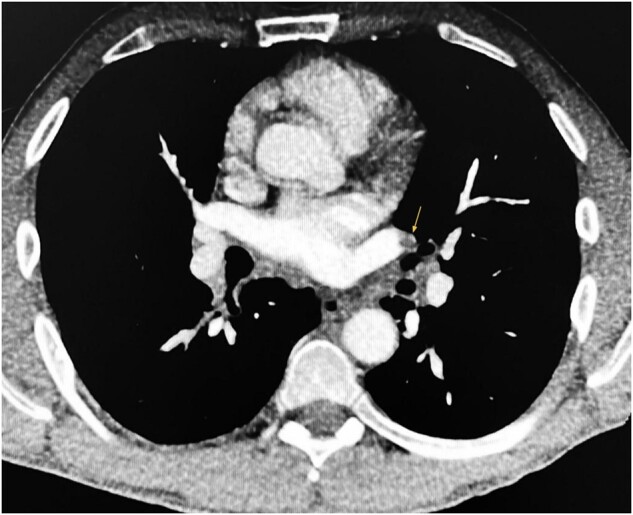
Contrast-enhanced chest CT scan showing pulmonary embolism in the left pulmonary artery (arrow).

**Figure 3:**
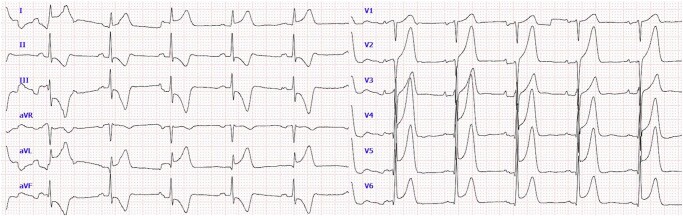
ECG indicating a diagnosis of antero-lateral ST-elevation myocardial infarction.

A 48-year-old man presented to our hub emergency department with complaints of headache and visual impairment that had occurred the previous day. Physical examination revealed a left superior quadrantanopia, with no other acute neurological findings. Blood pressure was 145/75 mmHg, HR 77 b.p.m., and SatO2 99% on air. An electrocardiogram (EKG) showed sinus rhythm. Except for mild anaemia (haemoglobin at 10.5 g/dL), all other laboratory chemical values were within normal limits. A non-contrast brain CT scan displayed hypodensity in the right inferior occipital lobe, indicating a subacute ischaemic stroke (*Figure 1A*). The patient was subsequently admitted to the Stroke Unit, where aspirin 300 mg was administered. The following day, a contrast-enhanced CT scan of the head and neck revealed an occlusion of the right posterior cerebral artery (P3), likely of embolic origin. Interestingly, the scan also revealed occlusion of one branch of the superior lobar artery in the left lung, raising suspicion of concomitant pulmonary embolism (PE). To confirm the diagnosis, the CT scan was extended to include the entire chest, which confirmed the presence of PE also involving the homolateral proximal part (*Figure 2*). Additionally, an 8 mm suspicious pulmonary nodule was detected in the right inferior lobe. Considering the patient’s haemodynamic stability and low-risk profile, enoxaparin 8000 IU twice daily was initiated. On the second day in the Stroke Unit, the patient suddenly developed severe chest pain accompanied by diaphoresis. The EKG (*Figure 3*) indicated an anterior STEMI, leading to the urgent transfer of the patient to the catheterization lab. Despite the emergency situation, the underlying cause of the critical scenario remained undetermined. Differential diagnoses included patent foramen ovale resulting in both pulmonary and systemic embolism, acquired hyper-coagulability possibly related to a paraneoplastic syndrome, and COVID-19 infection.

## Investigation and management

**Figure 4:**
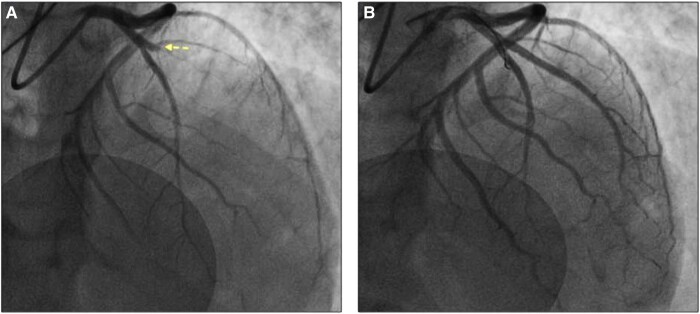
The coronary angiogram revealed an occlusion in one of the two left anterior descending arteries (dual LAD anatomy) (*A*). Manual thromboaspiration was performed and a 3.5 × 22 mm stent was successfully deployed (*B*).

The coronary angiography (*Figure 4A*) showed an occlusion of one of the two left anterior descending arteries (dual LAD anatomy), with all other coronary arteries being patent. Manual thromboaspiration (Export, Medtronic Vascular, USA) was performed and the lesion was pre-dilated with a 3 mm non-compliant balloon. Then, a 3.5 mm, 22 mm long zotarolimus-eluting stent was deployed, with final Thrombolysis in Myocardial Infarction (TIMI) flow of 3 (*Figure 4B*). The patient was haemodynamically stable throughout the procedure, without significant arrhythmic events.

Despite the high thrombotic risk due to the recent ischaemic stroke, PE, and STEMI, the patient also presented a high bleeding risk according to the ARC-HBR criteria (Hb < 11 g/dL, active malignancy, ischaemic stroke).^4^ Furthermore, there was a significant risk of haemorrhagic transformation of the ischaemic stroke.

Thus, we chose to administer cangrelor bolus (30 μg/kg) and maintenance (4 μg/kg/min) during the procedure, to have better control over the dual antiplatelet therapy (DAPT) by virtue of the rapid offset of cangrelor in case of development of major intracerebral haemorrhagic sequelae. Thus, for further safety, we therefore decided to continue cangrelor at a reduced dosage of 0.75 μg/kg/min for an additional 12 h. The patient was finally admitted to the coronary care unit. Echocardiogram showed reduced left ventricular ejection fraction (45%) with hypokinesis of the middle and distal anterior wall. No other significant abnormalities were found. He was started with ramipril 2.5 mg, pantoprazole 20 mg, and atorvastatin 80 mg. After three brief runs of non-sustained ventricular tachycardia, bisoprolol 2.5 mg was also added to the therapy. The patient remained asymptomatic during the cangrelor infusion and no new neurological signs appeared. We repeated a brain CT scan at 12 h (*Figure 1B*) from PCI that did not reveal haemorrhagic transformation or other radiological complications of the recent stroke. We therefore decided to transition from cangrelor (total infusion duration at a reduced dose: 12 h) to clopidogrel (loading dose of 300 mg plus 75 mg/day). The remainder of the patient’s stay in the coronary intensive care unit was free of complications. Before the hospital discharge, a CT scan of the abdomen revealed a pancreatic mass (highly suspicious for malignancy), and multiple liver metastasis. CA19.9 was extremely high. The patient was transferred to the oncology department on triple antithrombotic therapy (TAT) with aspirin 100 mg s.i.d., clopidogrel 75 mg s.i.d., and enoxaparin 8000 UI b.i.d. on board and chemotherapy with gemcitabine and cyclophosphamide was begun. The patient came one month after discharge for the first follow-up visit at our post-myocardial infarction outpatient clinic. No major bleeding complications were reported. Aspirin was interrupted and he was advised to continue clopidogrel and apixaban (10 mg b.i.d. for the first 7 days followed by 5 mg b.i.d.).

## Discussion

We have presented a challenging scenario due to the temporal concurrence of ischaemic stroke, PE, and STEMI. Pulmonary embolism was diagnosed as an incidental finding, and enoxaparin was preferred to the direct oral anticoagulants due to the ongoing oncologic diagnostic process (biopsy). When STEMI occurred, the need for a DAPT added up, giving rise to serious concerns for the balance between ischaemic (suspicious of cancer, the recent ischaemic stroke, the PE, and superimposed STEMI) and bleeding (ARC-HBR criteria: Hb < 11 g/dL, active malignancy, ischaemic stroke) risks.^4^ Thus, a drug-eluting stent approved for a short duration of DAPT was chosen (i.e. Resolute Onyx, Medtronic) to safely interrupt the TAT early and continue with enoxaparin and a P2Y12 receptor inhibitor only.^5^ Then, cangrelor was chosen as a second antiplatelet agent in the acute phase, better to monitor a possible haemorrhagic transformation of the ischaemic stroke.

This non-thienopyridine i.v. ATP analogue is characterized by rapid, potent, and reversible P2Y12 inhibitory effects.^6^ Moreover, cangrelor showed superior efficacy in reducing thrombotic complications compared with clopidogrel in patients undergoing PCI,^7^ and its efficacy was assessed in three large randomized controlled trials, CHAMPION PCI,^2^ CHAMPION PLATFORM,^3^ and CHAMPION PHOENIX.^7^ In the registration trials, cangrelor was administered as a bolus of 30 μg/kg followed by a 4 μg/kg/min infusion over 2 h or the duration of the procedure (whichever longer); on the other hand, results from the BRIDGE trial, where cangrelor was used in patients awaiting coronary artery bypass grafting,^8^ identified the optimal regimen to be 0.75 μg/kg/min in terms of antiplatelet blockage and major bleeding events. Interestingly, 15.1% of them had presented with STEMI.^8^ Besides the indications mentioned above, observational data also exist on the addition of a reduced dosage of 0.75 μg/kg/min for several hours to maintain the rapid offset properties during the early periprocedural period, with a low incidence of mild-to-moderate bleeding events and no severe, life-threatening, or intracranial bleeding.^9^ However, its usage is off-labelled and not yet approved by the principal drug agencies.

In our patient, the prolonged cangrelor infusion at 0.75 μg/kg/min proved effective and safe, as the brain CT scan performed after almost 12 h of continuous infusion revealed no haemorrhagic transformation of the ischaemic stroke nor new bleeding complications. It is of particular interest since the main trials^2,3,6,7^ excluded patients with recent (<1 year) ischaemic stroke and with malignancy.^2,3,6,7^

Thus, this case highlights cangrelor’s efficacy and safety profile even in challenging scenarios characterized by high haemorrhagic and thrombotic risk and not included in the major guidelines, possibly broadening its clinical indication.

## Conclusion

Prolonged cangrelor bridging at a reduced dose of 0.75 μg/kg/min may represent an effective and safe option in acute patients requiring P2Y12 inhibition and presenting both high ischaemic and high bleeding risks.

## Data Availability

The data underlying this article are available in the article.

## Lead author biography

Federico Oliveri is a young Italian cardiologist who graduated from the University of Pavia. Under the mentorship of Professor Leonardi, he developed a keen interest in antiplatelet therapy. Presently, he is pursuing a fellowship in Interventional Cardiology and a PhD at the Leiden University Medical Center (LUMC) in the Netherlands, with his research primarily focused on the treatment of calcified coronary lesions.

**Consent**: In compliance with the guidelines set forth by the Committee on Publication Ethics (COPE), we hereby confirm that the patient involved in this study has been adequately informed about the nature of the research, the usage of their data, and their rights to privacy and confidentiality. Written informed consent was obtained, ensuring their agreement to participate and for the use of their data in this publication. All efforts have been made to protect patient identity and personal details in accordance with ethical standards and patient privacy rights.

**Funding:** The present article received no external funding.
